# Patterns of Sedentary Behavior among Older Adults in Care Facilities: A Scoping Review

**DOI:** 10.3390/ijerph18052710

**Published:** 2021-03-08

**Authors:** Kin-Chung Wilson Leung, Kim-Wai Raymond Sum, Yi-Jian Yang

**Affiliations:** 1 Department of Sports Science and Physical Education, The Chinese University of Hong Kong (CUHK), Hong Kong, China; wilsonleung@cuhk.edu.hk (K.-C.W.L.); kwsum@cuhk.edu.hk (K.-W.R.S.); 2 CUHK Jockey Club Institute of Ageing, The Chinese University of Hong Kong, Hong Kong, China

**Keywords:** elderly, mobility, sedentariness, inactivity, sitting, lying, residential aged care, retirement communities, care homes, institutional

## Abstract

Understanding the sedentary patterns can guide the design of strategies to engage older adults in physical activity. This scoping review aimed to synthesize available evidence on sedentary behaviors in care facilities. We searched PubMed/MEDLINE and Web of Science for studies published from inception through October 2020. Eighteen studies were included and reviewed according to the Preferred Reporting Items for Systematic reviews and Meta-Analyses (PRISMA) guidelines. Data obtained were analyzed based on levels of care provided. Overall, daily sedentary time was higher among residents in high level care facilities (e.g., nursing homes) (11.6 h/day) than intermediate/mixed level care facilities (e.g., assisted living) (9.5 h/day). In intermediate/mixed level care facilities, television (TV) viewing was the most common sedentary activity (2.5–2.9 h/day; 26% of daily sedentary time), while napping was the most favorite sedentary activity (4.7 h/day; 36% of waking hours) in high level care facilities. Sex differences in daily patterns of sedentary behavior (sedentary time, uninterrupted bouts, and bout durations) were commonly observed in intermediate/mixed level care facilities, as exemplified by men being more sedentary by 0.7–1.1 h/day. In summary, this study highlights distinctive sedentary patterns among older adults residing in different levels of care facilities, addressing a pressing need for customized interventions to engage care facility residents in physical activity.

## 1. Introduction

Due to an increase in life expectancy, the elderly population has increased on an unprecedented scale and is estimated to ascend from 900 million (12%) in 2015 to 2 billion (22%) in 2050 worldwide [1]. In some places where people have the longest longevity (e.g., Hong Kong and Japan), the proportion of adults aged 65 years or older is projected to exceed 30% by 2050 [2]. To meet the challenges brought by such demographic changes, the demand for residential or nursing care is estimated to double by 2050 [3].

Care facilities can be divided based on the level of care provided [4]. Intermediate level care facilities (e.g., assisted living facilities) are usually independent housing units providing a level of care that is greater than community-living, but less than skilled nursing care in nursing homes [5]. These facilities cater to older people with certain levels of disabilities and provide services in support of their activities of daily living (ADLs), including personal and supportive care services, recreational activities, meals, housekeeping, laundry, and transportation. In the United States, almost 40% of older people admitted to assisted living facilities were dependent on at least three ADLs, especially bathing, meal preparation, and medication management [6,7]. Compared to intermediate care, high level care facilities (e.g., nursing homes) provide inpatient nursing and rehabilitative services in addition to personal care for long-term care residents [8]. A meta-analysis on 77 population-based studies across 12 data sources revealed that three or more ADL dependencies, cognitive impairment, and prior nursing home use were strong predictors of nursing home admission [6]. Moreover, some facilities provide both of these levels of care, for example, continuing care retirement communities, which are campus-like communities with independent living, assisted living, and skilled nursing care available on site in order to meet a continuum of ageing care needs.

In care facilities, older adults are generally physically inactive for most of their awake time, largely owing to pre-existing physical limitations and ADL assistance. It was shown that older adults in care facilities spent 79% of their awake time sedentary, 20% on low-intensity physical activity, and only 1% on moderate-to-vigorous physical activity [9]. Of note, only a few residents met the WHO physical activity guidelines for adults aged 65 years and above, as made evident by the fact that none of them performed moderate-to-vigorous physical activity continuously in bouts of at least 10 min duration and only less than 10% accumulated over 30 min of moderate-to-vigorous physical activity per day [9]. The impacts of care settings on residents’ physical activity levels and functional status were also evident as, after adjusting for treatment status and baseline differences, assisted living residents normally declined in physical activity levels and functions over a 6-month length of stay, yet nursing home residents conversely revealed an increase in physical activity levels and functions [7]. For care facility residents who are typically frailer and have low levels of fitness, Barber and colleagues (2015) [9] suggested that replacing sedentary activities with low-intensity physical activity could be more effective than focusing on promoting moderate-to-vigorous physical activity at the beginning, to engage the residents in an active lifestyle. Thus, limiting sedentary behavior may serve as a first step to achieving successful ageing, of which a key component is sustained physical functioning subsequent to physical activity [10].

Sedentary behavior is defined as any waking activities that cost low levels of energy expenditure [i.e., 1.0–1.5 metabolic equivalents] while in a sitting, reclining, or lying posture [11]. A systematic review on 22 population-based studies across more than 10 countries showed that community-dwelling older adults spent 9.4 h sedentary per day on average (equating to 65–80% of their waking time), while a self-report of sedentary time was lower, with an average of 5.3 h daily [12]. According to both objective (e.g., using accelerometers) and subjective measurements, men were more likely to be sedentary than women, with an average difference ranging from 9 to 30 min. However, heterogeneity of measurement and reporting methodologies across studies made results neither comparable nor easily synthesized. Moreover, variations in sedentary behavior across weekdays versus weekend days in the ageing population remain poorly understood [13]. Recently, systematic reviews showed that a reduced amount or interrupting prolonged bouts of sedentary time (i.e., more breaks) independent of physical activity levels was favorably associated with many geriatric-relevant health outcomes, especially all-cause mortality [14] and physical performance [15]. Considering the habitual pattern of total sedentary time and bouts, as well as no apparent day-to-day sedentary time compensation among older adults [16], interventions by breaking up prolonged periods of sedentary behavior seem effective in bringing about positive habitual changes in senior living.

Given that previous reviews on sedentary behavior have mostly been conducted in either community-dwelling [12] or mixed-setting older populations [14,15,17], how sedentary they are and how sedentary time is accumulated, specifically among older adults in care facilities, are still not rigorously reviewed. Results obtained from these earlier reviews may not be generalized to older adults in care facility settings, as the level of physical and cognitive impairment in the care facility aged populations is typically greater than older adults residing in the community [18,19,20]. According to expert consensus [21], there were also limited studies conducted to highlight the impact of care facility environments on sedentary time accumulation. Therefore, reviewing evidence of sedentary behavior in care facilities has become a research imperative. Therefore, the objective of this scoping review was to determine daily patterns (e.g., hours, types, number and duration of uninterrupted bouts, step count, and weekday-to-weekend trends) of sedentary behaviors among older adults in care facilities by synthesizing the results based on different levels of care. Given the sex differences in motivation and compliance to exercise interventions [22,23], falling patterns [24,25], and age-related muscle weakness and gait/balance deficit [26] that may affect mobility patterns of older men and women differently, we also aimed to identify sex differences in sedentary behavior patterns in care facilities. Collectively, an improved understanding of patterns and specific domains of sedentary behavior could guide the design of strategies to engage older adults in physical activity in care facility settings.

## 2. Materials and Methods

This review followed the Preferred Reporting Items for Systematic reviews and Meta-Analyses (PRISMA) guidelines. The study protocol was developed by the first author (K.-C.W.L.) based on the PRISMA extension for the Scoping Reviews Checklist [27]. Prior to data collection, the protocol was approved by the coauthors (K.-W.R.S. and Y.-J.Y.). Reporting of the study flow and findings was in accordance with the PRISMA statement [28]. 

### 2.1. Eligibility Criteria

To address the objectives of this scoping review, observational studies (e.g., cross-sectional, prospective, retrospective, case-control, or cohort) were the focus in our review. The inclusion criteria were: (1) text availability: full text, (2) language: English, (3) participant and setting: older adults residing in care facilities, (4) article type: peer-reviewed original research, and (5) study purpose and design: not necessarily just to specifically examine the amount or specific domains of sedentary behavior, but sedentary behavior reported as part of the results was also considered. For intervention studies, only baseline data were summarized. Qualitative studies, laboratory studies, or studies other than original research (e.g., reviews, meta-analyses, study protocols, comments, letters, case reports, and guidelines) were excluded. 

### 2.2. Information Sources

Studies published in the databases of PubMed/MEDLINE and Web of Science were searched from inception through October 2020. The Text Word terms used in the search (Title/Abstract/Subject/Keywords) included sedentary behavior, sedentary lifestyle*, sedentariness, sitting, lying, older, elder*, ageing, aging, senior*, senior citizen*, and geriatric. Since the definition and classification of care facilities are state-bound and vary across countries [5], we conducted literature search using a broader range of Text Word terms, including assisted living, assisted facilit*, residential care, residential facilit*, residential home*, residential aged care, care facilit*, care home*, institutional*, nursing facilit*, nursing home*, nursing care, retirement communit*, home* for the aged, long-term care, and long term care. The search queries for each database are summarized in the Supplementary File. 

### 2.3. Search Strategy 

Eligibility of the searched articles was initially screened by the first author (K.-C.W.L.) using a three-stage approach to review the title, abstract, and full text. In brief, the initial screening of the publications was based on the title, followed by the abstract. Exclusion criteria for the initial screening were clustered into the study (not an original research article or qualitative study, and unrelated study objectives) and participant (irrelevant study participants and not care facility settings) levels. The initial screening process adopted a stepwise approach; for instance, if the searched publication was an original yet qualitative study, the publication would be immediately excluded, with no other criteria being considered. Once the information was not confirmed in the initial screening on the title and abstract (e.g., whether the studies were conducted in care facility settings), full-text assessment for such a publication, along with other eligible studies, would be conducted. To avoid missing any relevant papers, the reference lists of the searched articles or review papers were also reviewed. All duplicates were removed when publications were screened. All included studies were finally checked for relevancy by the coauthors (K.-W.R.S. and Y.-J.Y.). 

### 2.4. Data Collection

Data extracted from the included studies were sedentary behavior (measurement, definition, and daily patterns (i.e., total time, types, number and duration of uninterrupted bouts, weekday-to-weekend total time variations, and sex differences)), levels of care (intermediate, mixed, or high), age (range and mean), number of participants, and sex distribution. In line with the framework or guidance for scoping reviews, as previously described [29,30], methodological quality assessment of the included studies was not undertaken.

### 2.5. Synthesis of Results

Since patterns and measures of sedentary behavior varied substantially across studies, total sedentary time or time for specific domains of sedentary behavior was transformed into mean hours per day to ensure comparability of results. The percentage of daily sedentary time relative to waking time was summarized, and the weighted average of sedentary time across care facilities in different levels of care was computed relative to the sample size of each included study. The daily numbers of uninterrupted sedentary bouts (e.g., 10+, 20+, 30+, 40+, 50+, and 60+ min) were also reported. Moreover, significant findings regarding weekday-to-weekend variations and sex differences in the patterns of sedentary behavior were summarized. All findings were then tabulated, followed by a narrative summary describing how the findings were related to the study objective. Finally, two independent investigators (K.-W.R.S. and Y.-J.Y.) checked and edited each entry for accuracy and consistency. 

## 3. Results

### 3.1. Study Selection

**Figure 1:**
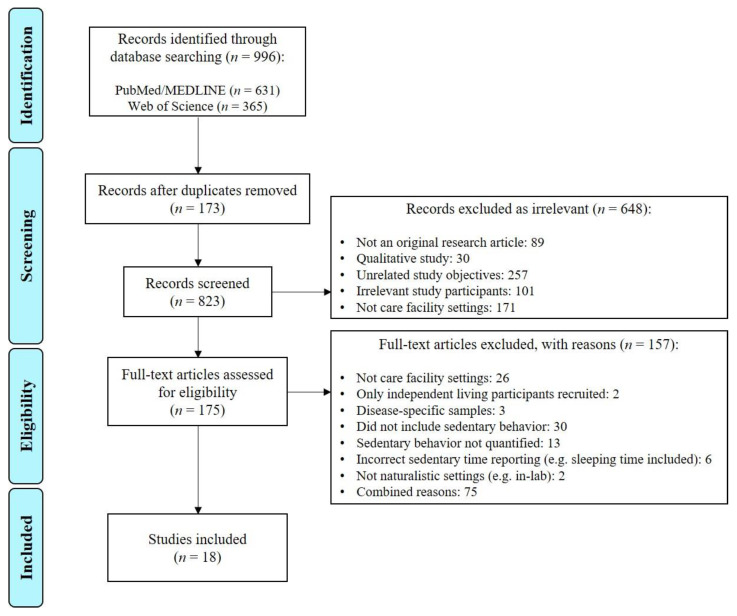
Flow chart of the publication selection process.

The database search yielded a total of 996 records (631 from MEDLINE and 365 from Web of Science), and 823 publications were then included in the initial screening after duplicates were removed. After reviewing the titles and abstracts, 175 articles were included for full-text assessment. A total of 157 articles were then excluded due to study-related (not care facility settings (*n* = 26), only independent living participants recruited (*n* = 2), disease-specific samples (*n* = 3), or not naturalistic settings (e.g., in-lab) (*n* = 2)), reporting (did not include sedentary behavior (*n* = 30), sedentary behavior not quantified (*n* = 13), or incorrect sedentary time reporting (e.g., sleeping time included) (*n* = 6)), or combined reasons (*n* = 75). Altogether, 18 publications were finally included for review and narrative analysis after full text assessment. Figure 1 shows the selection process of the 18 included publications.

### 3.2. Study Characteristics 

The 18 included studies were published between 2002 to 2020 and conducted in seven countries, including The United States (*n* = 6) [31,32,33,34,35,36], Australia (*n* = 5) [37,38,39,40,41], The United Kingdom (*n* = 2) [9,42], Canada (*n* = 2) [18,43], The Netherlands (*n* = 1) [44], and two countries (The United Kingdom and Spain) (*n* = 2) [45,46]. In total, there were 11 cross-sectional studies [9,18,32,33,35,36,37,38,39,41,42], three randomized controlled trials [31,44,45], two pre-post intervention studies [34,43], one prospective cohort study [40], and one participatory action research [46], with sample sizes ranging from 9 to 230. Eleven studies were conducted in either intermediate [9,18,34,36,42,43,45,46] or mixed [31,33,44] level care facilities, while seven studies were conducted in high level care facilities [32,35,37,38,39,40,41]. The participants in intermediate/mixed and high level care facilities were mainly women (42–90% and 65–85%, respectively), and their respective ages ranged from 64–101 and 61–100 years. Characteristics of the 18 included studies are summarized in Table 1. 

### 3.3. Sedentary Behavior Measurements

There were three methods commonly used for measuring sedentary behavior in care facilities (e.g., objective, self-reported, and naturalistic observation). Ten studies reported objectively-measured sedentary behavior using either accelerometers [9,18,35,38,39,42,44,45,46] or pedometers [31], five studies used self-reported questionnaires for measuring sedentary behavior [34,36,37,40,41], and two studies reported sedentary behavior using both objective and self-reported methods [33,43] (Table 2 and Table 3). Moreover, one study adopted naturalistic observation conducted by trained observers based on body postures and activity types to examine sedentary behavior in care facilities [32] (Table 2). 

The accelerometer-based instruments used across studies included ActiGraph/MTI [9,18,33,35,38,42,44] and ActiPAL [39,43,45,46] (Table 2). One study measured sedentary behavior using digital pedometers (e.g., Fitbit Ultra activity tracker) [31]. The monitoring period varied from 2 to 9 days, with one week being the most common recording period [9,18,31,39,43,45]. 

The questionnaires commonly used for measuring sedentary behavior in care facilities were the Sedentary Behavior Questionnaire (SBQ) [33,34,36,43] and the International Physical Activity Questionnaire (IPAQ)-Short Form [37,40,41] (Table 3). One study used a modified version of the SBQ, which added more age-relevant sedentary behavior examples under each category of sedentary behavior (e.g., hobbies: doing crafts, playing bingo, etc.) [33]. 

One study adopted a behavioral observation approach for measuring sedentary behavior in a naturalistic setting [32] (Table 2). Specifically, sedentary behavior was defined by body postures, including standing upright, sitting, and reclining, and recorded at 5-min intervals from 8:00 to bedtime (i.e., 13 h). Meanwhile, the context (e.g., location and activity), where the sedentary time was accumulated, was recorded (e.g., watching TV, reading, napping, socializing, personal care activities, movement, etc.). 

### 3.4. Patterns of Sedentary Behavior Based on Levels of Care

**Figure 2:**
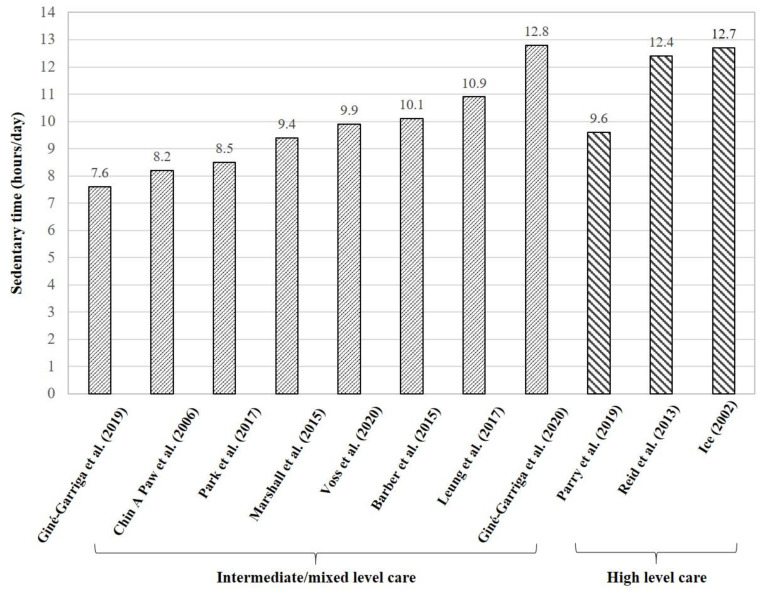
Daily sedentary time measured by accelerometers and naturalistic observations in care facilities.

Owing to the fact that care facility residents are less amenable to self-report because of pre-existing cognitive impairments [18,19], only daily sedentary time/behavior measured by accelerometers/pedometers and naturalistic observation was included for comparisons across intermediate/mixed and high level care facilities. Daily sedentary time in intermediate/mixed and high level care facilities was 7.6–12.8 h/day (71–87% of waking time) [9,18,33,42,43,44,45,46] and 9.6–12.7 h/day (85–98% of waking time) [32,38,39], respectively (Table 2). The weighted average of sedentary time was higher among residents in high level care facilities (11.6 h/day) [32,38,39] than intermediate/mixed level care facilities (9.5 h/day) [9,18,33,42,43,44,45,46] (Figure 2). Moreover, residents in high level care facilities (729 ± 321 steps/day) [31] had fewer daily step counts than those in intermediate/mixed level care facilities (4556 ± 2624 steps/day) [35] (Table 2).

In intermediate/mixed level care facilities, watching TV was self-reported to be the most common sedentary activity (2.5–2.9 h/day), followed by computer use (1.2–2.4 h/day), reading (1.5–1.9 h/day), and talking with friends (1.4–1.8 h/day) [33,36,43] (Table 3). Screen activities (TV viewing and computer use) accounted for 35% of daily sedentary time [36]. In high level care facilities, behavioral observations in a naturalistic setting showed that napping was the most favorite sedentary activity (4.7 h/day; 36% of waking time), followed by doing nothing (2.1 h/day; 16% of waking time) and watching TV (0.4 h/day; 3% of waking time) [32] (Table 2).

### 3.5. Weekday-to-Weekend Trends in Sedentary Behavior

Thus far, there was no study conducted to identify weekday/weekends trends in sedentary behavior in high level care facilities. Therefore, only findings generated in intermediate level care facilities were summarized. Overall, accelerometer-measured sedentary time did not differ significantly between weekdays and weekends [9,33,46] (Table 2), but self-reported sedentary time was higher on weekdays than weekends by 0.7–1.1 h/day [33,34] (Table 3). A similar yet non-significant weekday/weekend trend in sedentary time (weekdays: 14.6 h; weekend days: 11.0 h) was also self-reported [43]. In particular, the residents spent more time on computer use by 0.2 h/day, talking/thinking by 0.4 h/day, and group activities by 0.5 h/day during weekdays versus weekends [33].

### 3.6. Sex Differences in Patterns of Sedentary Behavior

**Figure 3:**
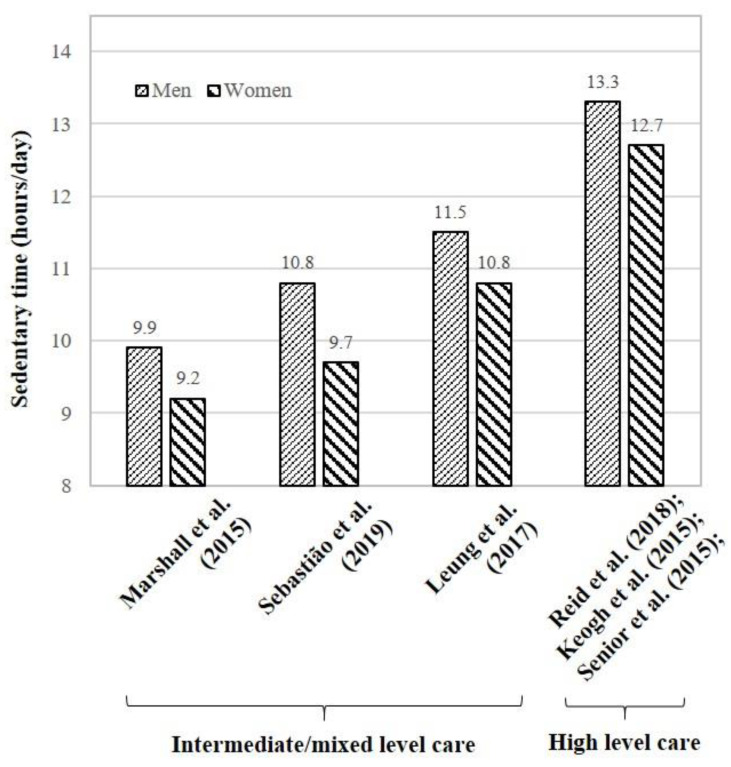
Sex differences in daily sedentary time in care facilities. In intermediate/mixed level care facilities, Marshall et al. (2015) and Leung et al. (2017) consistently showed that, as measured by accelerometers, men were more sedentary than women by 0.7 h/day. Similarly, Sebastião et al. (2019) found that a self-report of daily sedentary time was higher in men than women by 1.1 h/day. However, in high level care facilities, there was no significant difference in daily sedentary time measured by self-reported questionnaires between older men and women.

In intermediate/mixed level care facilities, accelerometer-based measurements showed that men were more sedentary than women by 0.7 h/day [18,33] (Figure 3). Additionally, Men (41.2 ± 35.3 bouts) had fewer bouts of sedentary behavior than women (52.7 ± 22.1 bouts), but the average duration of each bout was longer in men (17.1 ± 38.4 min) than women (12.3 ± 7.8 min) [18]. Likewise, self-reported sedentary time was shown to be higher in men than women by 1.1 h/day [36] (Figure 3). However, in high level care facilities, there was no significant difference in daily sedentary time between older men (13.3 ± 2.3 h/day) and women (12.7 ± 3.3 h/day) [37,40,41].

In intermediate/mixed level care facilities, women spent less on hobby activities by 0.6 h/day and daytime napping by 0.3 h/day, yet computer use demonstrated mixed findings across studies [33,36].

## 4. Discussion

To the best of our knowledge, this is the first review to synthesize knowledge concerning patterns of sedentary behavior among older adults in care facilities. This study found that older adults in high level care facilities (e.g., nursing homes or long-term care) were more sedentary than those in intermediate/mixed level facilities (e.g., assisted living) in terms of higher sedentary time [9,18,33,38,39,42,43,44,45,46] and fewer step counts [31,35] per day. This is likely owing to the fact that most residents who are admitted to high level care facilities commonly have more severe chronic conditions that adversely affect their mobility patterns, as over half of nursing home residents (50–96%) require staff (i.e., assisted transfer) and/or mechanical assistance (i.e., dependent transfer) to stand or move [32,47]. In addition, lack of motivation for physical activity interventions in long-term care, as exemplified by low recreational activity participation rates (6–31%), could explain such high levels of sedentary behavior [23].

Evidence from current systematic reviews revealed a causal relationship between sedentary behavior and health outcomes in older people [14,15]. Therefore, engaging care home residents in meaningful activities throughout the day is of utmost importance. Our findings showed that watching television (TV) (2.5–2.9 h/day) was the most common sedentary activity among residents in intermediate/mixed level care facilities [33,36,43]. Unfortunately, daily TV viewing time longer than 2 h was prospectively associated with a higher likelihood of suffering agility limitations and incident frailty in older adults by 18–25% and 10–47%, respectively [48]. On the other hand, findings generated by naturalistic observations showed that residents in high level care facilities spent most of their awake time on napping (36%; 4.7 h/day) and only 3% of the time on watching TV [32]. The author explained that the residents were usually parked in front of the TV, yet they were indeed sleeping. Although TV (location) was recorded 19% of the waking time, watching TV as an activity was only 3% of the time. This reflects that the residents are not too interested in watching TV and the reason why they spent much time on TV is that they may not have adequate choices of activity or may be less motivated to participate in other activities. Most importantly, intervening daytime sleepiness is imperative because excessive daytime napping (>2 h/day) is commonly associated with chronic sleep complaints and higher likelihood of suffering severe cognitive impairments (e.g., dementia) in older adults [49]. Regarding activity choices, a recent scoping review of 39 randomized controlled trials provided strong to moderate evidence about the therapeutic effectiveness of some recreational activities, for example, Tai Chi, walking, dancing, and ball games, in improving physical performance among residents in long-term care [23]. We particularly recommend Tai Chi and ball games for high level care residents because these two exercises can be performed in a sitting posture that is more suitable for those with compromised physical functioning. This review also highlighted a number of motivators for recreational activity program delivery in care facilities, including clear instruction, staff encouragement, attendance documentation, and minimal equipment. In addition, according to the WHO guidelines, older adults of 65 years or above are recommended to participate in household chores in order to meet the recommended levels of physical activity for health [50]. Due to physical limitations and lower levels of fitness, care facility residents are encouraged to participate in physically-affordable household activities, especially gardening, which was shown to cost adequate energy expenditure [51] and hold promising therapeutic value for frail and pre-frail nursing home residents in terms of perceived happiness [52,53], ADLs [53], and interpersonal intimacy [53]. In respect of changes in care facility environments, Pomeroy et al. (2011) [35] suggested that simple modification of indoor and outdoor environmental barriers (e.g., improved lighting, active living signs, workout stations, greenery provisions, etc.) was sufficient enough to improve functions and physical activity levels of nursing home residents. Of note, appropriate lighting can have awakening effects on older adults [49], so residents can participate in other activities in lieu of excessive napping.

In intermediate level care facilities, there was no apparent discrepancy in accelerometer-measured sedentary time between weekdays and weekends [9,46], but self-reported sedentary time was higher on weekdays than weekends [33,34,43]. Since most older adults after retirement do not engage in work- and school-related commitments, daily sedentary time across weekdays and weekends may not vary substantially. The mixed findings could be due to cognitive reliance of self-report; in care facilities, older adults commonly have cognitive defects and are incapable of accurately recalling their daily activity patterns. This can also explain why Marshall et al. (2015) [33] only observed sex differences in accelerometer-measured, but not self-reported, daily sedentary time among care facility residents. However, activity measurements by accelerometers do not provide information about the context in which sedentary time is accrued (e.g., TV viewing, computer use, reading, and socializing). Considering the limitations of both accelerometer-based and self-reported measures, naturalistic observation by trained researchers can be complementarily used to provide information about different types of sedentary behaviors and assess the accuracy of data obtained in respect of its feasibility in both high and intermediate level care facilities [32,54]. Furthermore, a novel technology coupled with wearable cameras is currently being developed to objectively obtain contextual information about sedentary behavior (e.g., TV viewing) in care facilities [55].

Both objective and self-reported measurements consistently reported that men were more sedentary than women in intermediate/mixed level care facilities [18,33,36]. The sex differences in sedentary duration may be attributed to the fact that men were less likely (20–30%) to participate in organized activities than women, as evidenced by over two-third of participants being women in care facilities [22,23]. Additionally, frequency of falls was higher in older men than women under similar clinical conditions [56], and older men who frequently fell were more likely to have reduced recreational/leisure activity and household/yard work [57]. In addition, different family priorities and responsibilities may explain why men were more sedentary than women. With a frail spouse, women are more likely than men to spend their time on caregiving, engendering less sedentary pastime [58]. Given recent research showing that breaking up prolonged sedentary bouts could confer favorable health outcomes (e.g., less likely to suffer abdominal obesity) on older women but not older men [59], altering sedentary patterns by organizing recreational activities tailored to older men and women should be guaranteed. For instance, older men tend to gain more from strenuous activities in respect of physical and psychosocial correlates, whereas older women are more likely to benefit from lighter activities with a strong socializing element (e.g., walking) [60]. Nonetheless, there was no sex difference in patterns of sedentary behavior in high level care facilities [37,40,41]. This is likely due to the fact that most residents in high level care facilities (e.g., nursing homes) are much frailer and unable to stand or move without staff and/or mechanical assistance (i.e., assisted or dependent transfer) (50–96%) [32,47]. As their mobility patterns are greatly shaped by the daily routine of care facilities, sex differences in patterns of sedentary behavior were, therefore, not commonly observed in high level care facilities.

Nevertheless, our findings need to be interpreted with caution due to the following study limitations. First, limited studies on the patterns of sedentary behavior (e.g., number of uninterrupted bouts, bout durations, weekday-to-weekend trends) have been conducted in high-level care facilities, making our results less comparable to intermediate level care settings. Second, the present review could not confirm the causal relationship between sedentary behavior and health outcomes among care facility residents. This is largely as a result of a limited number of relevant evidences and susceptibility of bias among the included studies, which are mostly non-experimental descriptive studies (e.g., 12 out of 18 studies are correlational and case-control studies) [61]. Third, there were only three studies explicitly explaining the time spent in sedentary behavior at resident level in intermediate/mixed level care facilities [9,18,36], while only one relevant study was conducted in high level care settings [41]. Of note, these four studies were all cross-sectional in nature, rendering weak evidence in supporting the causal linkage. Therefore, owing to the limited number and bias susceptibility of the relevant evidence [61], the underlying causes for an inactive lifestyle in care facilities remain poorly understood. Fourth, none of the included studies were conducted in Asia. This greatly limits the generalizability of our results extending to care facilities in Asian countries, where the contexts (e.g., ageing rates, institutionalization rates, support ratio, etc.) may differ considerably from Western countries [62]. Future research on the impacts of care facility environment on sedentary behaviors among Asian older adults should be warranted. Finally, the database searching for and scanning of eligible studies were mainly conducted by one single researcher (the first author). To reduce biases, the first author carried out the search and scanning in duplicate with at least one week apart. Similar results were eventually reproduced, and the results were finally verified by two independent researchers.

## 5. Conclusions

This scoping review is the first to highlight distinctive sedentary patterns among older adults residing in different levels of care facilities. In intermediate/mixed level care facilities, sex differences in daily patterns of sedentary behavior (sedentary time, uninterrupted bouts, and bout durations) were apparently observed, underscoring a pressing need for sex-specific motivational strategies to further mobilize older adults in this setting. More specifically, interventions targeting older men are urgently needed as men are more vulnerable than women to a sedentary lifestyle. For high level care residents, we highly recommend Tai Chi and ball games, which are empirically proven and can be customized (e.g., seated exercises) in order to cater to the needs of residents who commonly have functional impairments. Improved lighting and residents’ participation in simple household chores (e.g., gardening) are equally important to improve the current situation of low physical activity in high level care facilities. Given that little is known about the health impacts of a sedentary lifestyle on care facility residents, future intervention trials examining the effects of reducing sedentary time on geriatric-relevant health outcomes (e.g., physical function, cognitive function, urinary incontinence, and mental health (e.g., depressive symptoms)) [21] are also highly deserved in an effort to inform better sedentary behavior interventions in care facilities.

Since the improvement of the quality of life of older adults serves as an opportunity to ‘bring together governments, civil society, international agencies, professionals, academia, the media, and the private sector for ten years of concerted, catalytic and collaborative action’ [63], the results of this scoping review provide insightful research and health promotion ideas to different stakeholders to support the development of active environments in care facilities and physical activity interventions for older adults. In particular, physical activity intervention throughout the life course can contribute to sustained independence and improved population health and well-being [64]. Taken together, physical health is a lifelong endeavor and stakeholders should be reminded that older adults have the right to appropriate physical activity options and implement interventions to improve physical activity levels [65].

## Figures and Tables

**Table 1:** Characteristics of the included studies published from 2002 to 2020 (*n* = 18).

	Total (*n* = 18)	Intermediate/Mixed (*n* = 11)	High (*n* = 7)
**Number of Study (%)**			
**Country**			
United States	6 (33.3)	4 (36.4)	2 (28.6)
Australia	5 (27.8)	---	5 (71.4)
United Kingdom	2 (11.1)	2 (18.2)	---
Canada	2 (11.1)	2 (18.2)	---
The Netherlands	1 (5.6)	1 (9.1)	---
Two countries (United Kingdom and Spain)	2 (11.1)	2 (18.2)	---
**Study design**			
Cross-sectional	11 (61.1)	5 (45.5)	6 (85.7)
Prospective cohort	1 (5.6)	---	1 (14.3)
Randomized controlled trial	3 (16.7)	3 (27.3)	---
Pre-post intervention	2 (11.1)	2 (18.2)	---
Participatory action research	1 (5.6)	1 (9.1)	---
**Study participants**			
Age (years) ^a^	61–101	64–101	61–100
Total number	1059	848	211
Sample size ^a^	9–230	9–230	27–98
% women ^a^	42–90%	42–90%	65–85%

^a^ Data were presented as range.

**Table 2:** Objectively-measured sedentary behavior among older adults in care facilities (*n* = 13).

Author, Country, and Study Design	Level of Care	Age (Years)		Participants	Sedentary Behavior			
		Range	Mean		Type	Measurement	Definition	Daily Pattern
Giné-Garriga et al. (2020); United Kingdom and Spain; randomized controlled trial ^a^ [45]	Intermediate	n/a	82.9 ± 13.6	*n* = 31 52% women	Sedentary time	ActivPAL3 monitor; thigh-worn; measurement: 7 days, waking time	Sitting and lying postures	12.8 ± 3.4 h
Voss et al. (2020); Canada; pre-post intervention ^a^ [43]	Intermediate	n/a	82.2 ± 8.7	*n* = 9 90% women	Sitting time	ActivPAL4 inclinometer; thigh-worn; measurement: 7 days, 24 h	Sitting and lying postures	9.9 ± 3.0 h; bouts >30 min: 6.3 ± 2.8 h
Giné-Garriga et al. (2019); United Kingdom and Spain; participatory action research [46]	Intermediate	n/a	83.2 ± 11.6	*n* = 22 59% women	Sedentary time	ActivPAL monitor; thigh-worn; measurement: 9 days, waking time	Sitting and lying postures	Weekday: 7.6 ± 3.2 h Weekend: 7.7 ± 2.9 h
Leung et al. (2017); Canada; cross-sectional [18]	Intermediate	≥65	86.7 ± 7.5	*n* = 114 85% women	Sedentary time	ActiGraph GT1M; waist-worn; measurement: 7 days, waking time	<100 cpm	10.9 ± 1.1 h (87% of waking time); 52 bouts with each lasting 13 min on average
Park et al. (2017); United Kingdom; cross-sectional [42]	Intermediate	65–99	77.5 ± 8.2	*n* = 85 68% women	Sedentary time	ActiGraph GT3X+; hip-worn; valid data: 10 h/day, ≥3 days, with 1 weekend day	<100 cpm	8.5 ± 1.8 h (71% of waking time)
Barber et al. (2015); United Kingdom; cross-sectional [9]	Intermediate	>65	82.6 ± 9.2	*n* = 33 64% women	Sedentary time	ActiGraph GT3X; hip-worn; measurement: 7 days, waking time	<100 cpm	10.1 ± 2.2 h (79% of waking time) 3.3 ± 1.3 bouts of ≥60 min with each lasting 2.8 ± 3.5 h on average
Harkins et al. (2017); United States; randomized controlled trial ^a^ [31]	Mixed	≥65	80.3 ± 5.9	*n* = 94 74% women	Step count	Fitbit Ultra activity tracker/pedometer; waist-worn;measurement: 7 days	<5000 steps	4556 ± 2624 steps
Marshall et al. (2015); United States; cross-sectional [33]	Mixed	n/a	83.5 ± 6.5	*n* = 230 70% women	Sedentary time	ActiGraph GT3X+; hip-worn; measurement: 6 days, waking time	<100 cpm	9.4 ± 1.5 h
Chin A Paw et al. (2006); The Netherlands; randomized controlled trial ^a^ [44]	Mixed	64–94	81.0 ± 5.6	*n* = 118 80% women	Sitting time	MTI model 7164; hip-worn; measurement: 3 days, waking time	<100 cpm	8.2 h
Parry et al. (2019); Australia; cross-sectional [38]	High	≥65	83.1 ± 8.6	*n* = 28 71% women	Sedentary time	ActiGraph GT3X; hip-worn; measurement: 5 days, waking time	<100 cpm	9.6 ± 1.5 h (85% of waking time); bouts >30 min: 5.3 ± 0.8 h (55% of total sedentary time)
Reid et al. (2013); Australia; cross-sectional [39]	High	61–96	84.2	*n* = 31 65% women	Sitting and lying time	activPAL3; thigh-worn; measurement: 7 days, 24 h	Sitting and lying postures	12.4 ± 1.7 h (85% of waking time); sitting time accrued in bouts ≥30 and ≥60 min: 73% and 44%, respectively
Pomeroy et al. (2011); United States; cross-sectional [35]	High	74–100	87.0 ± 6.7	*n* = 27 78% women	Step count	ActiGraph; measurement: 48 h	<5000 steps	729 ± 321 steps
Ice (2002); United States; cross-sectional [32]	High	65–100	87	*n* = 27 85% women	Sedentary time	Naturalistic observation; measurement: 1 day, waking time	Sitting and lying postures	12.7 h (98% of waking time)
								- Napping: 4.7 h - Nothing: 2.1 h - Watching TV: 0.4 h

^a^ Only baseline data were summarized. Abbreviations: counts per minute (cpm); television (TV). n/a: information was not provided by the articles.

**Table 3:** Self-reported sedentary behavior among older adults in care facilities (*n* = 7).

Author, Country, and Study Design	Level of Care	Age (years)		Participants	Sedentary Behavior			
		Range	Mean		Type	Measurement	Definition	Daily Pattern
Voss et al. (2020); Canada; pre-post intervention ^a^ [43]	Intermediate	n/a	82.2 ± 8.7	*n* = 9 90% women	Sedentary time and behavior	SBQ	Time spent on 10 sedentary activities ^c^	Weekday: 14.6 ± 3.9 h - Watching TV: 2.9 ± 1.7 h - Computer: 2.4 ± 2.1 h - Hobby: 1.9 ± 1.4 h - Talking: 1.8 ± 1.6 h - Reading: 1.7 ± 1.4 h - Listening to music: 1.4 ± 1.6 h - Napping: 0.7 ± 0.6 h - Church/theater: 0.6 ± 0.7 h - Transportation: 0.4 ± 0.5 h - Working: 0.1 ± 0.2 h Weekend: 11.0 ± 4.0 h - Watching TV: 2.7 ± 1.7 h - Computer: 1.7 ± 1.7 h - Reading: 1.5 ± 0.9 h - Talking: 1.4 ± 1.4 h - Hobby: 1.3 ± 1.4 h - Napping: 0.7 ± 0.6 h - Listening to music: 0.6 ± 1.0 h - Working: 0.6 ± 1.0 h - Transportation: 0.5 ± 0.4 h - Church/theater: 0.4 ± 0.7 h
Naber et al. (2019); United States; pre-post intervention ^a^ [34]	Intermediate	76–101	89.0 ± 6.6	*n* = 12 42% women	Sedentary time and behavior	SBQ	Time spent on 10 sedentary activities ^c^	Weekday: 6.0 h Weekend: 5.3 h
Sebastião et al. (2019); United States; cross-sectional [36]	Intermediate	≥60	84.7 ± 6.3	*n* = 100 70% women	Sedentary time and behavior	SBQ	Time spent on 10 sedentary activities ^c^	10.0 ± 0.0 h - Watching TV: 26% - Reading: 19% - Computer use: 12% Screen activities ^d^: 35% Non-screen activities ^d^: 65%
Marshall et al. (2015); United States; cross-sectional [33]	Mixed	n/a	83.5 ± 6.5	*n* = 230 70% women	Sedentary time and behavior	Modified SBQ	Time spent on 10 sedentary activities ^c^, with more age relevant sedentary activity examples under each category	11.4 ± 4.9 h - Watching TV/DVDs: 2.5 ± 1.5 h - Reading: 1.8 ± 1.1 h - Talking/thinking: 1.5 ± 1.5 h - Computer: 1.4 ± 1.4 h - Group activities: 1.2 ± 1.0 h - Hobby: 0.9 ± 1.0 h - Transportation: 0.7 ± 0.6 h - Napping: 0.5 ± 0.5 h - Others: 0.9 ± 1.2 h
Reid et al. (2018) ^b^; Australia; prospective ^a^ [40] Keogh et al. (2015) ^b^; Australia; cross-sectional [37] Senior et al. (2015) ^b^; Australia; cross-sectional [41]	High	≥60	84.5 ± 8.2	*n* = 98 71% women	Sitting time	IPAQ-Short Form	Sitting time on a week day in the last 7 days	12.9 ± 3.0 h

^a^ Only baseline data were summarized. ^b^ These three studies (Reid et al., 2018; Keogh et al., 2015; Senior et al., 2015), which all met the inclusion criteria, were conducted on the same cohort with the same baseline measures, so only one dataset was summarized. ^c^ Sedentary activities included napping, reading, listening to music, watching TV, computer, working, hobby, talking with friends, transportation, and church/theater. ^d^ Screen activities were TV viewing and computer use, whereas non-screen activities included napping, reading, listening to music, working, hobby, talking with friends, transportation, and church/theater. Abbreviations: Digital versatile discs (DVDs); International Physical Activity Questionnaire (IPAQ); Sedentary Behavior Questionnaire (SBQ); television (TV).

## Data Availability

Not applicable.

## Supplementary Materials

The following are available online at https://www.mdpi.com/1660-4601/18/5/2710/s1, Supplementary File: Search Strategy.
