# Supplementary material for: Patterns of Sedentary Behavior among Older Adults in Care Facilities: A Scoping Review
Source: Int J Environ Res Public Health. 2021 Mar 8;18(5):2710. doi: 10.3390/ijerph18052710 (PMC7967434; doi:10.3390/ijerph18052710)
Supplement: Supplementary file 1 [file ijerph-18-02710-s001.pdf]

Supplementary File

PubMed (MEDLINE) Search Strategy

("assisted living"[All Fields] OR "assisted facilit*"[All Fields] OR "residential care"[All Fields] OR "residential facilit*"[All Fields] OR "residential home*"[All Fields] OR "residential aged care"[All Fields] OR "care facilit*"[All Fields] OR "care home*"[All Fields] OR "institutional*"[All Fields] OR "nursing facilit*"[All Fields] OR "nursing home*"[All Fields] OR "nursing care"[All Fields] OR "retirement communit*"[All Fields] OR "home* for the aged"[All Fields] OR "long-term care"[All Fields] OR "long term care"[All Fields])

AND

("sedentary"[All Fields] OR "sedentary behavior"[All Fields] OR "sedentary behaviour"[All Fields] OR "sedentary lifestyle*"[All Fields] OR "sedentariness"[All Fields] OR "sitting"[All Fields] OR "lying"[All Fields])

AND

("older"[All Fields] OR "elder*"[All Fields] OR "ageing"[All Fields] OR "aging"[All Fields] OR "senior*"[All Fields] OR "senior citizen*"[All Fields] OR "geriatric"[All Fields])

Limiters

- Text availability: Full text
- Species: Humans
- Language: English

Web of Science Search Strategy

“sedentary” OR “sedentary behavior” OR “sedentary behaviour” OR “sedentary lifestyle*” OR “sedentariness” OR “sitting” OR “lying”

AND

“assisted living” OR “assisted facilit*” OR “residential care” OR “residential facilit*” OR “residential home*” OR “residential aged care” OR “care facilit*” OR “care home*” OR “institutional*” OR “nursing facilit*” OR “nursing home*” OR “nursing care” OR “retirement communit*” OR “home* for the aged” OR “long-term care” OR “long term care”

AND

Topic = “older” OR “elder*” OR “ageing” OR “aging” OR “senior*” OR “senior citizen*” OR “geriatric”

Limiters

- Document Types: ARTICLE
- Languages: English
